# Observation of giant Goos-Hänchen and angular shifts at designed metasurfaces

**DOI:** 10.1038/srep19319

**Published:** 2016-01-13

**Authors:** Venkata Jayasurya Yallapragada, Ajith P. Ravishankar, Gajendra L. Mulay, Girish S. Agarwal, Venu Gopal Achanta

**Affiliations:** 1 DCMP & MS, Tata Institute of Fundamental Research, Homi Bhabha Road, Mumbai 400005 INDIA; 2 Department of Physics, Oklahoma State University, Stillwater, OK 74078, USA

## Abstract

Metasurfaces with sub-wavelength features are useful in modulating the phase, amplitude or polarization of electromagnetic fields. While several applications are reported for light manipulation and control, the sharp phase changes would be useful in enhancing the beam shifts at reflection from a metasurface. In designed periodic patterns on metal film, at surface plasmon resonance, we demonstrate Goos-Hanchen shift of the order of 70 times the incident wavelength and the angular shifts of several hundred microradians. We have designed the patterns using rigorous coupled wave analysis (RCWA) together with S-matrices and have used a complete vector theory to calculate the shifts as well as demonstrate a versatile experimental setup to directly measure the shifts. The giant shifts demonstrated could prove to be useful in enhancing the sensitivity of experiments ranging from atomic force microscopy to gravitational wave detection.

Metasurfaces are 2-dimensional equivalents of metamaterials with features smaller than the wavelength specifically designed to modulate the phase, amplitude or polarization of electromagnetic field. The reports so far covered designer metasurfaces for flat optics to flexible metasurfaces, nonlinear and active metasurfaces that can be controlled, anisotropic metasurfaces for dispersionless response, miniature cavities, antennas, waveguides, complex mode generation by introducing sub-wavelength features within the diffracting apertures among others covered in recent review articles^1^,^2^,^3^,^4^,^5^. Surfaces can also be designed to have sharp phase changes which effect the reflection of an optical beam. A beam reflected off an interface experiences spatial and angular shifts depending on the polarization and the beam profile. Spatial displacements are the well-known Goos-Hänchen (GH) shift in the plane of incidence and the Imbert-Fedorov shift normal to the plane of incidence^6^,^7^,^8^. Angular shifts that depend on the beam profile were recently demonstrated by Merano *et al.*^9^. Artmann calculated the GH shift by combining the Fresnel coefficients with the expressions for phase shift for a plane wave and showed that there is polarization dependence in the lateral shift^10^. This has been viewed as a consequence of the presence of an evanescent component of the field beyond the interface. More rigorous formulations have been proposed based on Poynting vector analysis, rigorous integrals, coherence matrices and angular spectrum representations^11^,^12^,^13^,^14^. Techniques used for reflection at plane interfaces have been extended to patterned surfaces by Guther and Kleeman who used an integral equation system method with parameterization of the periodic profile to calculate the shift and shape of the beams diffracted from a sinusoidal grating^15^,^16^. The angular shifts have not been studied much experimentally^9^, although a theoretical treatment^17^ for the beam reflected near the Brewster angle has existed for several decades.

Bretenaker *et al.*^18^ demonstrated a novel way of measuring the GH shift in single reflection by making use of the difference in the s- and p-polarized reflected beams. This was followed by the study of enhanced shifts at a Wood’s anomaly in a metallic grating^19^. This was further followed by the measurement of shifts at single reflection off metallic surfaces^20^. Yin *et al.* showed that large GH shifts exist at reflection from a metal (silver) surface when surface plasmons are excited^21^. Large shifts have also been demonstrated for self-confined light beams^22^. For the recent theoretical and experimental advances in this field, one may consult the review article by Bliokh and Aiello^23^.

Typically, the GH shift at a plane metallic interface is expected to be comparable to the wavelength of light. So, for any practical application, ways to enhance the shifts are needed. In general, enhancement is expected when there is a large modulation of the complex amplitude of the reflected field as is the case close to the critical angle in total internal reflection (TIR) and during the excitation of various surface modes. A recent renewal of interest in beam shifts resulted in the demonstration of weak measurement of shifts^24^,^25^,^26^, as well as applications in sensing and switching^27^,^28^. General theoretical models to calculate the shifts for incident beam of any polarization has been presented^29^, as well as a unified model applied to harmonic generation by which the second harmonic beam generated at a metallic interface is also shown to experience the shift^30^.

The sharply varying angular deviations of the beams which occur at such a resonance have potential applications in sensing^9^. In view of the recent work it would be interesting to design patterned surfaces that are optimum for such giant beam shifts. In the current report, we extend our general formulation of shifts to grating interfaces and angular shifts at surface plasmon resonance. We then proceed to demonstrate a robust high resolution experimental setup that can directly measure the plasmon enhanced shift of p-polarized beam with respect to an s-polarized reference beam.

## Results

### Design and fabrication of the gratings

**Figure 1:**
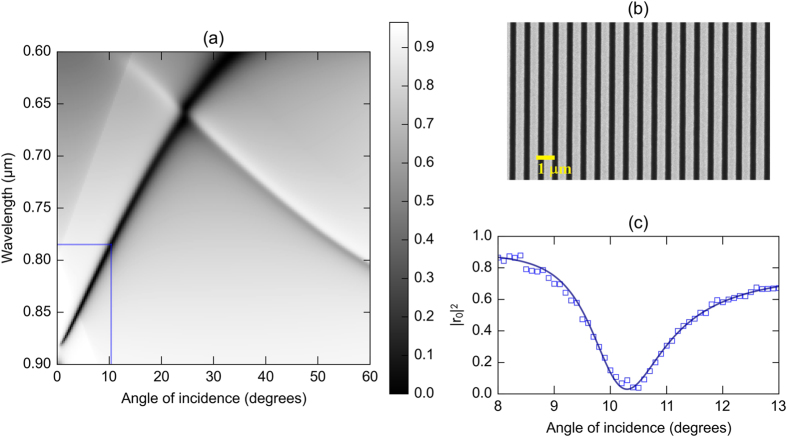
(**a**) A contour plot of the calculated specular reflectivity of the fabricated gratings at different incident wavelengths and angles of incidence. The dark band corresponds to the excitation of a SP mode at the interface. The blue lines indicate the position of the SP resonance for the wavelength (785 nm) used in the experiments. (**b**) An SEM image of the fabricated grating. It consists of PMMA stripes (dark) on a thick gold film (bright) on a Silicon substrate. The length of the yellow scale bar is 1 μm. (**c**) Measured reflectivity (blue squares) from the grating using light of wavelength 785 nm. The solid line shows the reflectivity calculated using RCWA.

We study a metasurface comprising of a large area (~4 mm^2^) polymethyl methacrylate (PMMA) gratings on an optically thick gold film (250 nm). The optical properties of these gratings were calculated using rigorous coupled wave analysis (RCWA)^31^,^32^,^33^. These calculations provide insight into the dispersion relation of various SPP modes that exist in the structure. We designed the grating configuration such that the SPP resonance occurs at 785 nm for an incidence angle of 10.3°. The specular reflectivity from the grating exhibits a strong dip associated with the SP resonance as shown in Fig. 1(a). RCWA has been used to calculate the reflected and transmitted field amplitudes in the air-grating interface given the complex field amplitude of incident plane wave and its polarization. The amplitudes can be used to compute the Goos-Hänchen shift as described in the following section. The optical properties of the fabricated grating metasurfaces (Fig. 1b) are in agreement with the predictions of our calculations (Fig. 1c).

### Calculation of the Goos-Hänchen shift

**Figure 2:**
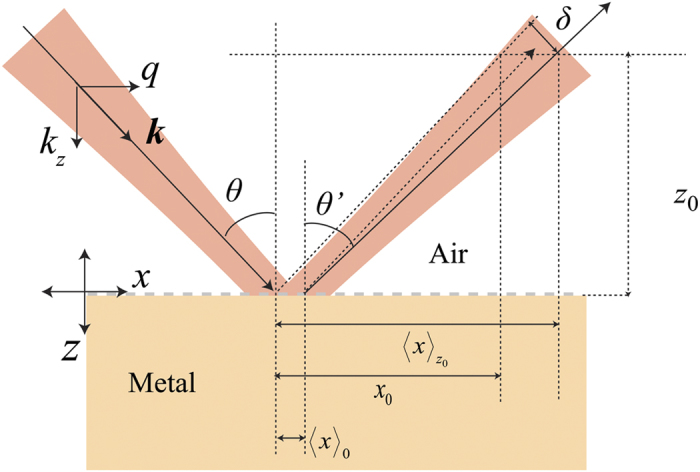
The geometry of the problem being studied. The spatial Goos-Hänchen shift measured on a detector is given by <x > cos(θ) (please see text for details). The total shift, however, involves contributions from angular deviations which result in significant displacements of the beam centroid as the reflected beam propagates.

The Goos-Hänchen shift is a consequence of the variation of the complex reflected amplitude across the wave vector distribution of the incident beam of finite spatial extent. It manifests itself as a displacement of the centroid of the reflected field distribution with respect to the incident field at the interface. The geometry of the problem is schematically shown in Fig. 2. In order to estimate the shift in the beam, we compute the location of this displaced centroid. This can be evaluated as follows. The incident beam is first resolved into its plane wave components.


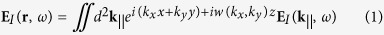


Here, 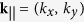 and 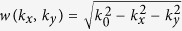, where 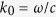. Following the approach given in refs 29 and 30, we consider the Fourier transform of a Gaussian beam whose field distribution has a centroid at x = 0 in the plane of the interface and propagates with a central wave-vector 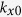. Such a field distribution is described by the following expression,


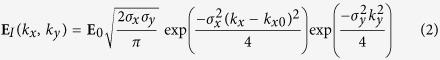


Here *σ*_*y*_ is the waist radius of the incident beam. Also, 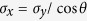 as a consequence of oblique incidence. The reflected field amplitudes can also be expressed in the form of Eq. (1) as,


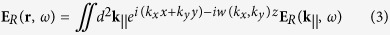


For a simple planar interface, one could use the Fresnel reflection formulae to obtain these reflected field amplitudes from the incident amplitudes. Transfer matrices provide a route of computation for stratified media and RCWA can be used for periodically patterned interfaces. Following computation of the reflected amplitude coefficients, the centroid of the reflected field distribution in a plane parallel to the interface and positioned at *z* = *z*_0_ can be calculated. A full treatment of the spatial and angular shifts is presented in the supplementary information which results in the following expression that includes both the spatial and angular shifts.


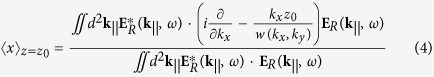


The above expression for the shift in the centroid of the beam in the plane of incidence consists of two parts. Each of them is estimated by numerical integration. The first is the spatial GH shift which is equal to the total shift at *z* = 0 and is given by,


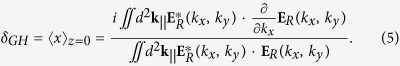


**Figure 3:**
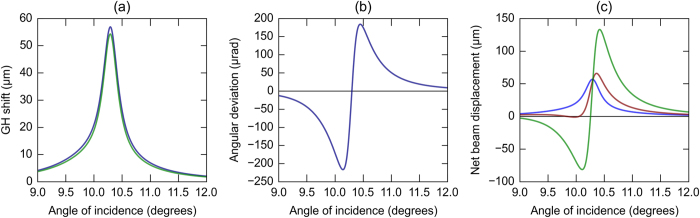
Calculated beam shifts. (**a**) The spatial GH shift (blue) at SP resonance at the grating interface for an incident wavelength of 785 nm, gently focused to a waist size of ~160 μm at the interface. The green curve shows the result of Artmann’s formula. (**b**) The angular deviation (*θ′− θ*) from the expected angle of reflection. (**c**) Resultant beam shift calculated for the beam parameters in (**b**) at propagation distances 0 cm (blue), 12.8 cm (red) and 52.8 cm (green) along the reflected beam path.

This expression reduces to Artmann’s result for slowly varying phases^10^. This is in agreement with the intuitive understanding that the shifts tend to be largest when there is a steep variation of the phase of the reflected field with respect to the angle of incidence. This typically occurs at the surface plasmon resonance condition for interfaces involving metals. However, there can be deviations for focused beams at sharp resonances (see Fig. 3a)^34^.

The second part in Eq. (4) is the angular shift. This occurs if there exists a reflectivity gradient across the k-space spread of the incident beam. As a result, for a beam with an angular spectrum of finite width, plane wave components corresponding to different angles of incidence experience different reflectivities within the beam. This results in a change in the direction of the reflected beam. This is more pronounced in situations where the change in reflectivity is sharp such as at a surface plasmon resonance. Such effects have been discussed by Guther and Kleemann^15^,^16^, and also demonstrated experimentally by Merano *et al.*^20^. The modified angle of reflection *θ′* is given by,


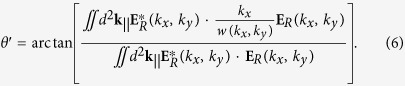


The deviation from the expected angle of reflection (*θ*′ *− θ*) varies with the angle of incidence as shown in Fig. 3b. The displacement in the position of the beam centroid due to the angular deviation is proportional to the distance of propagation of the reflected beam. On propagating a sufficient distance, the angular contribution to the beam displacement tends to overwhelm the spatial GH shift contribution (Fig. 3c).

### Experimental studies of the GH shift

The most common way to measure GH shift has been to periodically switch between s- and p- polarization and measure the difference in position using a Quadrant Photodiode (QPD) and lock-in amplifier combination. While employing this method, however, one must be very careful that the reflected intensities for *s-* and *p-* polarizations are equal. If not, the signal will also include a contribution from the intensity difference between the s- and p- polarized reflections. Therefore, it would no longer be a faithful measurement of the beam shift. To overcome this limitation, Li *et al.* proposed the beamsplitter scanning method^35^.

**Figure 4:**
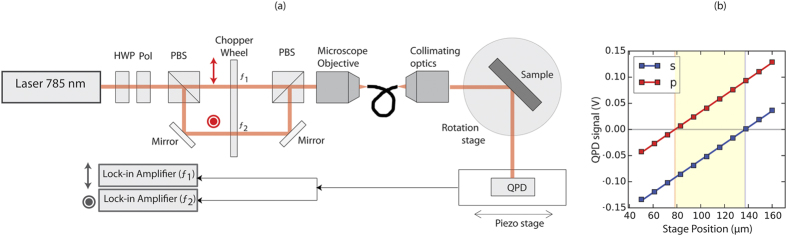
(**a**) The experimental setup for measuring the shifts. (**b**) Sample data acquired during one scan of the detector. The method of obtaining the x-intercepts using a straight line fit has been illustrated. The shaded yellow region (corresponding to the separation between the x-intercepts of s- and p-polarized beams) indicates the relative shift between the s- and p- polarizations.

We propose a novel measurement technique with high sensitivity as shown in the schematic of Fig. 4(a). In this technique, a position sensitive detector QPD is scanned across the cross section of the beam around its centroid and the difference signal from the detector is recorded at different detector positions. The measurements were carried out for both s- and p- polarizations simultaneously without otherwise altering the experimental configuration. By fitting a straight line to the data points measured in such a scan, one can accurately determine the x-intercept, which is the position of the beam centroid. Therefore, difference in the x-intercepts of the two straight lines is the difference between the GH shift for s- and p- polarizations (see Fig. 4b). The advantage of using such an approach is two-fold. Close to the centroid, the signal from the QPD is small, and electronic noise can affect the measurement significantly. Away from the centroid, however, the electronic noise is much smaller than the QPD signal. By extracting the x-intercept point from the straight line fit to the data, the accuracy of the measurement is considerably improved. Secondly, as detector is scanned across the centroid of the beam, changes due to variation of (and even difference between s- and p-) incident intensity on the detector only affect the slope of the straight line fits and not the position of the intercepts. Therefore, one does not need to calibrate the response of the QPD with respect to the incident intensity. This method has been found to be robust and insensitive to variations in the intensity.

As shown in Fig. 4a, we use a diode laser (Schafter + Kirchhoff LNC-56CM-785-35-B23-A8-H-6) operating at 785 nm. A half-wave plate followed by a polarizer have been used to control the intensity and linear polarization of light being used. The horizontal and vertical polarization components are then split using a polarizing beamsplitter (PBS). The two components are chopped at different frequencies using a double slot chopper wheel. The beams are then recombined using a second PBS, and the light is coupled into polarization maintaining optical fiber (Thor Labs P1-780PM-FC-1). The output from the optical fiber is then gently focused (waist size ≈160 μm) onto the grating structure mounted on a rotation stage. The beam waist is located at the air-grating interface. The shift is measured using a QPD module (New Focus 2901) mounted on a motorized stage (Newport MFA-PPD) or a piezo-stage (PiezoJena Tritor 400). The QPD output is split and connected to two lock-in amplifiers (Stanford Research Systems SR-830) locked to the two chopper frequencies. Using different modulation frequencies for the two polarizations, the centroid positions of both the *s-* and *p-* polarizations can be detected in a single scan of the detector. This has proved to be very effective in reducing the duration of data acquisition as well as in reducing the effects of beam drift (if any) during the measurements. The experimental setup also allows for measuring the beam intensity profiles using a camera, by blocking either the s- or p- polarized beams as required.

**Figure 5:**
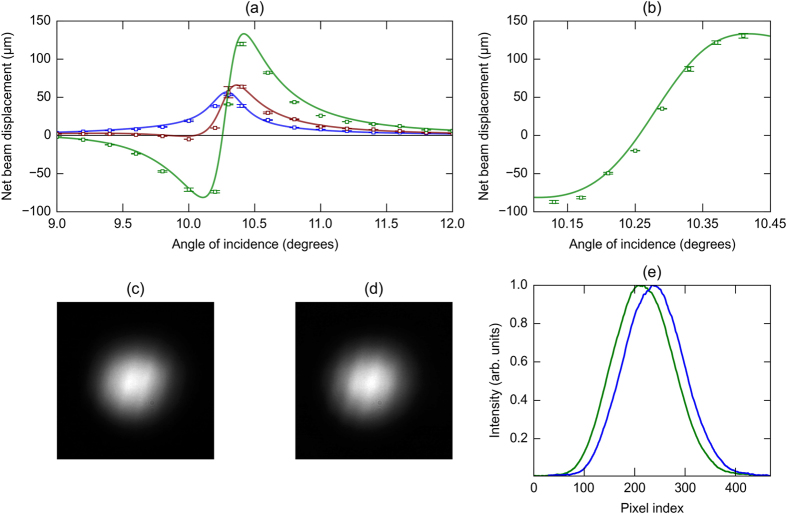
Experimental measurements of the beam shifts using the setup illustrated in Fig. 4. (**a**) Measurement of the total beam shift. Measured beam shifts with a collimated incident beam of light (blue) and with the incident beam gently focused to a beam waist ~160 μm at different propagation distances 12.8 cm (red) and 52.8 cm (green) of the reflected beam. Solid lines show the theoretical predictions in Fig. 3, and the symbols denote experimentally measured values. Error bars indicate the standard deviation of the set of measured values for each angle of incidence. (**b**) A magnified view of the behavior of the beam shift close to where the shift becomes zero. The very sharp variation of the beam position with a small change in the angle of incidence is clearly demonstrated. (**c**,**d**) CCD images of the beam profile of the reflected s- and p- polarized beams, respectively, close to the resonance at a propagation distance of approximately 12 cm. (**e**) The normalized beam profiles obtained by summing columns of the intensity data for s- (green) and p- (blue) polarized beams. The size of each pixel is 3.6 μm.

Experimental measurements of the shift, using this apparatus, are shown in Fig. 5a,b (expanded view near resonance). These measured shifts show good agreement with theoretical predictions (straight lines) after incorporating the effect due to the angular deviation. To illustrate the effects of the angular deviations and propagation of the reflected beam, measurements of the shift have been carried out at different distances from the grating. These features cause significant changes in the reflected beam centroid position when the beam is allowed to propagate over larger distances. These position changes are large enough to be easily observed using a simple CMOS camera (see Fig. 5c–e). It is interesting to note that above a certain propagation distance, there exists a point where the beam position matches with the the position of the reflected beam spot expected in case of shift-free Euclidean reflection. Operation around this point could prove to be useful in accurate sensing technologies.

## Discussion

At an angle of incidence of about 10 degrees, the transmitted +1 order excites a surface plasmon mode in the grating structure at 785 nm wavelength. This results in resonant coupling of the energy from the incident field to the SPP mode. The position of the resonance, as estimated from the dip in specular reflectivity, varies with incidence angle as shown in Fig. 1a for PMMA gratings on a gold surface. The large phase jumps in the reflected light associated with the resonance are expected to give rise to a large GH shift. This enhancement of the positive GH shift is evident from the experimentally measured values. There is an enhancement of the shift at resonance to upto 70 times the wavelength of the incident light. An advantage of such gratings is that one is able to obtain an enhanced GH shift at a variety of geometries and wavelengths, according to experimental convenience, by designing the grating suitably. This is in contrast to the prism configuration where the angle of incidence is necessarily close to the critical angle of incidence. The plasmon enhanced beam shifts reported here are comparable to those obtained in gratings at a Wood’s anomaly^19^.

Another aspect of functional nano-patterned interfaces, which is of significant utility, is their physically compact nature. To study the shifts at interfaces of small spatial extent, the incident beam needs to be focused in order to accommodate the entire cross section of the beam on the functional area. This leads to a spread in the wave vector values associated with such a beam. Within this spread, depending on the incidence angle relative to the resonant angle, the sharp nature of the plasmon resonance attenuates a particular section of the k_x_ distribution more than the other. This will result in a deviation of the direction of propagation of the reflected beam from the expected angle of reflection. Depending on the convergence angle of the incident beam this could have a significant effect on the beam shifts measured.

The angular deviation of the p-polarized reflected beam changes sign at the angle of incidence where the resonance occurs. It is particularly interesting to note that the change from a negative value to a positive value occurs over a very small range of the angle of incidence. In spite of this large change, the beam profile is not distorted significantly and retains its symmetric nature about the centre as indicated by the direct beam profile images shown in Fig. 5c,d.

It may be noted that, even with a position detector capable of measuring with a resolution of 1 μm, one would be able to measure angular deviations of the order of few tens of μrad in a straightforward manner. Such measurements can be performed without having the beam to propagate over large distances from the reflecting interface. The observed variations in the enhanced beam shifts occur over a small range of incidence angles and can be utilized in accurate surface plasmon resonance based sensing. Such sensitivity could also be useful in very sensitive beam deflection measurements like in an AFM or gravitation wave detection experiments (LIGO). Angular deviations in reflected beams can also play a role in nonlinear optical phenomena, such as degenerate four wave mixing in the reflection geometry.

## Methods

Using RCWA the eigen-modes of propagation are calculated in each layer of the structure and the S-matrix method is used to solve for the reflected field amplitudes. The method used is based on the procedure outlined in Refs 31 and 32. These amplitudes have been used to calculate the reflectivity and the beam shifts using Eqs (5) and (6). Optical constants of gold have been obtained by interpolating the Johnson-Christy data^36^ with a cubic spline. The refractive index of PMMA has been taken from the material datasheet provided by the manufacturer^37^.

To study these phenomena experimentally, gratings consisting of PMMA stripes on metal have been fabricated. A 250 nm thick gold film was sputtered on a 10 nm thick Chromium adhesion layer on Silicon substrate. Since the skin depth for 785 nm wavelength in Gold is about 26 nm, the layer effectively behaves like bulk gold and can be treated as a gold half-space. Then a 4 percent solution of PMMA in Anisole (MicroChem PMMA 495 A4) has been spin coated on it such that the thickness of the layer is about 133 nm. Following this, gratings of period 800 nm and groove width 420 nm have been patterned onto this layer using electron beam lithography using a Raith eLine system with fixed beam moving stage writing technique. Using this feature, a grating of dimension 2 mm × 2 mm could be exposed within a few hours. The parameters have been so chosen that there exists a strong surface plasmon resonance involving the +1 transmitted order at an angle of incidence of 10.3°. The grating parameters have been measured using SEM and AFM characterization and it has been ensured that the gratings are of a high quality.

## Conclusions

To summarize, patterned surfaces with sub-wavelength features would give rise to large beam shifts. A rigorous vector model for both spatial and angular beam shifts at gratings involving metallic interfaces has been presented. To demonstrate the utility of this formulation, we have designed and fabricated dielectric gratings on a gold surface. As a consequence of the sharp surface plasmon resonance in such gratings, considerable spatial and angular shifts in the reflected beams can be observed. To demonstrate these phenomena a robust experimental setup to measure the shift, employing a scanning position sensitive optical detector, has been demonstrated. We have shown that the properties of the spatial displacement and angular deviations in the plane of incidence are in good agreement with the formulation. The sharp change in the sign of the beam shift due to angular deviations of the reflected beam could be employed in high sensitive angle measurements.

## Additional Information

**How to cite this article**: Yallapragada, V. J. *et al.* Observation of giant Goos-Hänchen and angular shifts at designed metasurfaces. *Sci. Rep.* **6**, 19319; doi: 10.1038/srep19319 (2016).

## Supplementary Material

Supplementary Information
