# Erratum: Observation of giant Goos-Hänchen and angular shifts at designed metasurfaces

**DOI:** 10.1038/srep23835

**Published:** 2016-04-13

**Authors:** Venkata Jayasurya Yallapragada, Ajith P. Ravishankar, Gajendra L. Mulay, Girish S. Agarwal, Venu Gopal Achanta

Scientific Reports 6: Article number: 1931910.1038/srep19319; published online: 01132016; updated: 04132016

In the original version of this Article, the symbol “
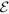
” in the equations was incorrectly written as “E”

In addition, the HTML version of the Article incorrectly listed Venkata Jayasurya Yallapragada, and not Venu Gopal Achanta, as corresponding author.

These errors have now been corrected in the PDF and HTML versions of the Article.

