# Supplementary material for: Observation of giant Goos-Hänchen and angular shifts at designed metasurfaces
Source: Sci Rep. 2016 Jan 13;6:19319. doi: 10.1038/srep19319 (PMC4725830; doi:10.1038/srep19319)
Supplement: Supplementary Information [file srep19319-s1.pdf]

Observation of giant Goos-Hänchen and angular shifts at designed metasurfaces

Venkata Jayasurya Yallapragada[1], Ajith P Ravishankar[1], Gajendra L Mulay[1], Girish S Agarwal[2], Venu Gopal Achanta[1]

[1] DCMP&MS, Tata Institute of Fundamental Research, Homi Bhabha Road, Mumbai 400005 India.
[2] Department of Physics, Oklahoma State University, Stillwater, OK 74078, USA.

A unified approach to the calculation of spatial and angular beam shifts

We begin by expressing the electric field profile of the reflected beam in terms of its various plane wave components.

$$\mathbf{E}_R(\mathbf{r},\omega) = \iint d^2\mathbf{k}_\parallel \; e^{i(k_x x + k_y y) - iw(k_x,k_y)z} \boldsymbol{\mathcal{E}}_R(\mathbf{k}_\parallel,\omega) \tag{1}$$

Here $\mathbf{k}_\parallel = (k_x, k_y)$ and $w(k_x,k_y) = \sqrt{k_0^2 - k_x^2 - k_y^2}$, where $k_0 = \frac{\omega}{c}$ is the magnitude of the free space wavevector of the light. To obtain the centroid of the beam in the x direction, we use the following.

$$\langle x \rangle = \frac{\iint dxdy \mathbf{E}_R^*(\mathbf{r},\omega) \cdot \mathbf{E}_R(\mathbf{r},\omega) x}{\iint dxdy \mathbf{E}_R^*(\mathbf{r},\omega) \cdot \mathbf{E}_R(\mathbf{r},\omega)} \tag{2}$$

Here, the reflected field amplitude components are obtained using Rigorous Coupled Wave Analysis (RCWA). We shall now proceed to simplify the numerator. Using Equation 1 we get

$$x\mathbf{E}_R = \iint d^2\mathbf{k}_\parallel \; x e^{i(k_x x + k_y y - iw(k_x,k_y)z)} \boldsymbol{\mathcal{E}}_R(\mathbf{k}_\parallel,\omega). \tag{3}$$

Here, we note that

$$\begin{aligned} -i\frac{\partial}{\partial k_x}\left[e^{i(k_x x + k_y y - iw(k_x,k_y)z)}\right] &= \left(x - z\frac{dw(k_x,k_y)}{dk_x}\right) e^{i(k_x x + k_y y - iw(k_x,k_y)z)} \\ &= \left(x + \frac{k_x z}{w(k_x,k_y)}\right) e^{i(k_x x + k_y y - iw(k_x,k_y)z)} \end{aligned} \tag{4}$$

Using the above result , Equation 3 can be rewritten as

$$x\mathbf{E}_R = \iint d^2\mathbf{k}_\parallel \; \boldsymbol{\mathcal{E}}_R(\mathbf{k}_\parallel,\omega) \left(-i\frac{\partial}{\partial k_x} - \frac{k_x z}{w(k_x,k_y)}\right) e^{i(k_x x + k_y y - iw(k_x,k_y)z)} \tag{5}$$

Using the above result, followed by a similar treatment for $\mathbf{E}^*_R(\mathbf{r},\omega)$, the numerator in Equation 2 can be expressed as

$$\iint dxdy \iint d^2\mathbf{k}_\parallel \iint d^2\mathbf{k}'_\parallel \; \Big\{ \boldsymbol{\mathcal{E}}^*_R(\mathbf{k}'_\parallel,\omega) e^{i(k'_x x + k'_y y - iw(k'_x,k'_y)z)} \cdot \boldsymbol{\mathcal{E}}_R(\mathbf{k}_\parallel,\omega) \left(-i\frac{\partial}{\partial k_x} - \frac{k_x z}{w(k_x,k_y)}\right) e^{i(k_x x + k_y y - iw(k_x,k_y)z)} \Big\}. \tag{6}$$

Using the result,

$$\int dx \int dy \; e^{i(k_x - k'_x)x + i(k_y - k'_y)y} = 4\pi^2 \delta(k_x - k'_x)\delta((k_y - k'_y)),$$

in Equation 6, followed by simplification, we obtain our final expression for the numerator in Equation 3, which is as follows.

$$\iint d^2\mathbf{k}_\parallel \; \boldsymbol{\mathcal{E}}^*_R(\mathbf{k}_\parallel,\omega) \cdot \left(i\frac{\partial}{\partial k_x} - \frac{k_x z}{w(k_x,k_y)}\right) \boldsymbol{\mathcal{E}}_R(\mathbf{k}_\parallel,\omega) \tag{7}$$

Similarly the denominator can be simplified into

$$\iint d^2\mathbf{k}_\parallel \; \boldsymbol{\mathcal{E}}^*_R(\mathbf{k}_\parallel,\omega) \cdot \boldsymbol{\mathcal{E}}_R(\mathbf{k}_\parallel,\omega). \tag{8}$$

The expression for the x position of the beam centroid in a plane parallel to the interface at $z = z_0$ is, therefore, the following.

$$\langle x \rangle_{z=z_0} = \frac{\iint d^2\mathbf{k}_\parallel \; \boldsymbol{\mathcal{E}}^*_R(\mathbf{k}_\parallel,\omega) \cdot \left(i\frac{\partial}{\partial k_x} - \frac{k_x z_0}{w(k_x,k_y)}\right) \boldsymbol{\mathcal{E}}_R(\mathbf{k}_\parallel,\omega)}{\iint d^2\mathbf{k}_\parallel \; \boldsymbol{\mathcal{E}}^*_R(\mathbf{k}_\parallel,\omega) \cdot \boldsymbol{\mathcal{E}}_R(\mathbf{k}_\parallel,\omega)} \tag{9}$$

The integrand in the numerator is a sum of two components. The first is the spatial Goos - Hänchen shift, which can be computed by substituting $z_0 = 0$ in Equation 9, and is as follows.

$$\delta_{GH} = \langle x \rangle_{z=0} = \frac{i \iint d^2\mathbf{k}_\parallel \; \boldsymbol{\mathcal{E}}^*_R(\mathbf{k}_\parallel,\omega) \cdot \frac{\partial}{\partial k_x} \boldsymbol{\mathcal{E}}_R(\mathbf{k}_\parallel,\omega)}{\iint d^2\mathbf{k}_\parallel \; \boldsymbol{\mathcal{E}}^*_R(\mathbf{k}_\parallel,\omega) \cdot \boldsymbol{\mathcal{E}}_R(\mathbf{k}_\parallel,\omega)} \tag{10}$$

At non-zero z_0, i.e. after allowing the reflected beam to propagate a certain distance, there is an additional contribution to the displacement of the centroid, which results from an angular deviation in the direction of the beam, given by,

$$\langle x \rangle_{z=z_0} = \frac{-\iint d^2\mathbf{k}_\parallel \; \boldsymbol{\mathcal{E}}^*_R(\mathbf{k}_\parallel,\omega) \cdot \frac{k_x z_0}{w(k_x,k_y)} \boldsymbol{\mathcal{E}}_R(\mathbf{k}_\parallel,\omega)}{\iint d^2\mathbf{k}_\parallel \; \boldsymbol{\mathcal{E}}^*_R(\mathbf{k}_\parallel,\omega) \cdot \boldsymbol{\mathcal{E}}_R(\mathbf{k}_\parallel,\omega)} \tag{11}$$

The reflected beam propagates at an angle θ' with the normal that is different from the expected angle of reflection (θ). This angle is given by

$$\theta' = \arctan\left[\frac{\iint d^2\mathbf{k}_\parallel\ \boldsymbol{\mathcal{E}}_R^*(\mathbf{k}_\parallel,\omega)\cdot\dfrac{k_x}{w(k_x,k_y)}\boldsymbol{\mathcal{E}}_R(\mathbf{k}_\parallel,\omega)}{\iint d^2\mathbf{k}_\parallel\ \boldsymbol{\mathcal{E}}_R^*(\mathbf{k}_\parallel,\omega)\cdot\boldsymbol{\mathcal{E}}_R(\mathbf{k}_\parallel,\omega)}\right]$$

from which it is straightforward to compute the angular deviation $\theta' - \theta$.
